# Cytotoxic Effects of Synthetic and Herbal Endodontic Irrigants on Human Red Blood Cells: An In Vitro Study

**DOI:** 10.7759/cureus.93522

**Published:** 2025-09-29

**Authors:** Panna Mangat, Bhaviya Chandel, Mampi Biswas, Sara Trivedy, Akshata Gupta, Nayan Shree, Seema Gupta

**Affiliations:** 1 Department of Conservative Dentistry and Endodontics, Kalka Dental College, Meerut, IND; 2 Department of Orthodontics, Kothiwal Dental College and Research Centre, Moradabad, IND

**Keywords:** chlorhexidine, cytotoxicity, garlic extract, herbal, irrigants, neem, sodium hypochlorite

## Abstract

Introduction: Endodontic irrigants are vital for disinfecting root canals; however, their potential to harm healthy cells drives the search for safer options. Herbal extracts may offer natural antimicrobial benefits with reduced toxicity compared with synthetic agents. This study aimed to evaluate the cytotoxicity of synthetic irrigants (5.25% sodium hypochlorite (NaOCl) and 2% chlorhexidine (CHX)) versus herbal options (neem and garlic extracts) on human red blood cells (RBCs) in vitro. The objectives of this study were to assess RBC viability across different concentrations to identify the least cytotoxic irrigant and evaluate neem and garlic extracts as biocompatible alternatives for endodontic therapy.

Materials and methods: This in vitro study was conducted at the Department of Conservative Dentistry and Endodontics, Kalka Dental College, Meerut, India. The study involved collecting 5 mL of venous blood from a healthy volunteer, followed by centrifugation at 1000 rpm for 10 minutes to isolate packed human RBCs. RBCs were washed with 0.9% saline and diluted to create a suspension, with 100 µL aliquoted into 164 test tubes per trial. Four experimental groups (5.25% NaOCl, 2% CHX, neem extract, and garlic extract) each had 40 tubes subdivided by irrigant volume (10-50 µL), and a saline control group had four tubes. Neem extract was prepared by boiling fresh leaves to a 25% concentration, whereas garlic extract was subjected to ethanol treatment and homogenization to 25%. After adding irrigants and incubating for three minutes, RBC viability was measured using an automated hematology analyzer. The experiment was repeated eight times to ensure reliability. Data were statistically analyzed. The tests used were the intraclass correlation (ICC) test and the Kruskal-Wallis test with Bonferroni post-hoc tests, with significance at p < 0.05.

Results: All groups showed significant ICC (p < 0.05), with NaOCl at 0.99 and neem at 0.58. Intergroup analysis revealed the lowest RBC viability for NaOCl (median = 3.26%), while neem (4.91%) and control (5.53%) showed the highest viability. Post-hoc tests confirmed the inferior performance of NaOCl (p < 0.01 vs. all). Concentration-dependent declines were significant across the groups (p < 0.001), with NaOCl showing the steepest drop. Within the concentrations, neem consistently outperformed the others.

Conclusion: Neem extract demonstrated superior biocompatibility as an endodontic irrigant, suggesting its potential as a safer alternative to synthetic agents, such as NaOCl.

## Introduction

Endodontic therapy, commonly known as root canal treatment, is a critical procedure in modern dentistry, aimed at eliminating microbial infections within the root canal system to preserve natural teeth [1]. A pivotal aspect of this treatment is the use of irrigating solutions, which play a vital role in disinfecting the root canal, dissolving organic debris, and flushing out residual bacteria [2]. The efficacy of endodontic irrigants lies in their antimicrobial properties, tissue-dissolving capabilities, and ability to penetrate complex root canal anatomies [3]. However, the biocompatibility of these irrigants is critical, as their cytotoxicity can adversely affect periapical tissues, including human red blood cells (RBCs), if extruded beyond the root apex during irrigation [4]. Therefore, ensuring the safety and biocompatibility of irrigants is paramount to prevent systemic or localized tissue damage and enhance the success of endodontic therapy.

Synthetic irrigants, such as 5.25% sodium hypochlorite (NaOCl) and 2% chlorhexidine (CHX), are widely used in endodontics owing to their potent antimicrobial and tissue-dissolving properties [5]. NaOCl is considered the gold standard irrigant because of its ability to dissolve necrotic tissue and eliminate a broad spectrum of microorganisms [6]. However, its high cytotoxicity, particularly at higher concentrations, raises concerns regarding potential damage to host tissues, including RBCs, which may lead to hemolysis or compromised cellular integrity [4,7]. Similarly, CHX, valued for its substantivity and antibacterial efficacy, may also exhibit cytotoxic effects, potentially affecting the structural and functional integrity of RBCs [8]. These concerns have prompted researchers to explore alternative irrigants that balance antimicrobial efficacy with biocompatibility.

In recent years, herbal irrigants have gained attention as potential alternatives to synthetic solutions because of their natural origin, perceived safety, and bioactive properties [9]. Neem (*Azadirachta indica*) extract, known for its antimicrobial, anti-inflammatory, and antioxidant properties, has shown promise for dental applications [9]. Its active compounds, such as nimbin and azadirachtin, may offer effective microbial control with reduced toxicity compared with synthetic irrigants [10]. Similarly, garlic (*Allium sativum*) extract, which contains allicin and other sulfur compounds, exhibits potent antibacterial and antifungal properties, making it a candidate for endodontic irrigation [11]. However, the cytotoxic effects of these herbal extracts on human RBCs remain underexplored, necessitating rigorous in vitro studies to evaluate their safety and suitability for clinical use. The cytotoxicity of endodontic irrigants on RBCs is a critical parameter because hemolysis or damage to RBCs can indicate potential toxicity to other host tissues [4].

This study aimed to evaluate and compare the cytotoxic effects of synthetic endodontic irrigants, specifically 5.25% NaOCl and 2% CHX, with herbal endodontic irrigants (neem extract and garlic extract) on human RBCs in an in vitro setting. The objectives were to compare the biocompatibility of synthetic versus herbal irrigants to identify the least cytotoxic option and explore neem and garlic extracts as biocompatible alternatives for endodontic therapy.

## Materials and methods

Study design

This in vitro experimental study was conducted in the Department of Conservative Dentistry and Endodontics at Kalka Dental College, Meerut, India, in a certified laboratory equipped with biosafety level 2 facilities to ensure sterile handling of biological samples and extracts. Ethical approval was obtained from the Institutional Ethical Committee (KDC/LTR/2023/0109) of the host institution, ensuring compliance with the ethical standards for human-derived samples and adhering to the principles of the Declaration of Helsinki.

Eligibility criteria

Informed consent was obtained from a single healthy volunteer, who was fully briefed on the study’s purpose, procedures, and minimal risks associated with blood donation, and who signed a consent form confirming voluntary participation. The participant, aged 18-35 years, was a non-smoker, non-alcoholic, and free from systemic diseases such as diabetes, hypertension, or anemia. The individual had no history of medication use, blood transfusions, or anticoagulant therapy in the preceding six months. Exclusion criteria included individuals with systemic illnesses, hematological disorders, pregnant or lactating women, and those unwilling to provide consent.

Sample size estimation

This study required a sample size of 160 to achieve 95% statistical power with a 5% alpha error level. An effect size of 0.34 was determined based on previous research examining cytotoxic effects on RBCs [12]. Using G*Power software version 3.1.9.2 (Heinrich Heine University, Düsseldorf, Germany), the sample size was calculated using an analysis of variance (ANOVA) omnibus fixed-effects model for four experimental groups. Each treatment group contained 40 samples, with an additional control group of four specimens included for comparative analysis.

Methodology

Blood was collected aseptically from 5 mL of venous blood using an ethylenediaminetetraacetic acid (EDTA) tube (BD Vacutainer®, Becton, Dickinson and Company, Franklin Lakes, NJ). The blood was centrifuged at 1000 rpm for 10 minutes using a centrifugal machine (Remi R-8C Laboratory Centrifuge, Remi Elektrotechnik Ltd., Mumbai, India) to separate plasma from packed RBCs. The packed RBCs were washed two to three times with 0.9% saline (Otsuka Pharmaceutical, Tokyo, Japan) to remove residual plasma, and centrifuged after each wash to ensure purity. A 5 mL RBC suspension was prepared by mixing 1 mL of packed RBCs with 4 mL of saline, and 100 µL of this diluted suspension was aliquoted into 164 test tubes per trial using a calibrated micropipette (Eppendorf, Hamburg, Germany).

**Figure 1:**
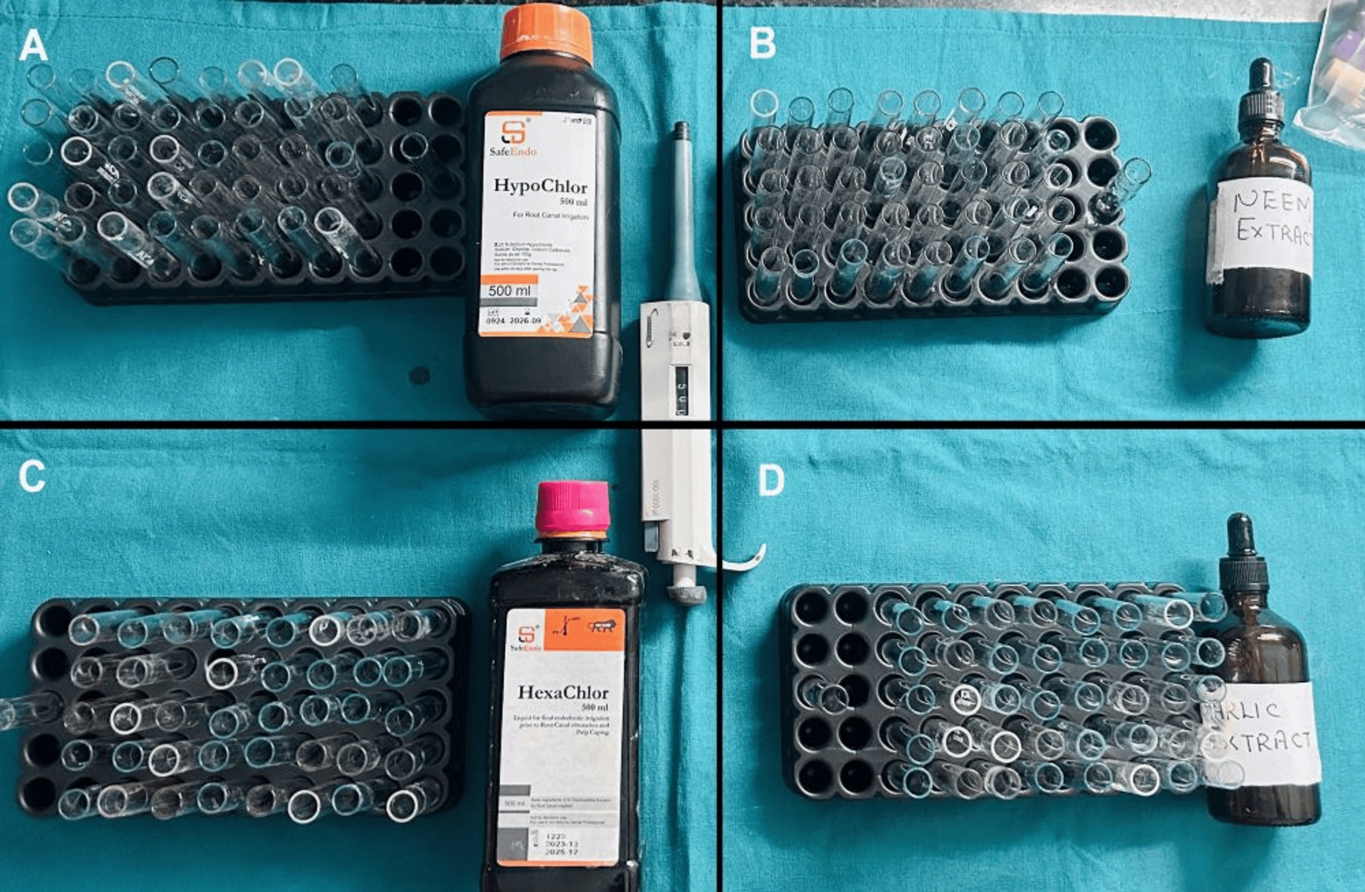
Experimental groups: (A) 5.25% sodium hypochlorite with 40 test tubes, (B) neem extract with 40 test tubes, (C) 2% chlorhexidine with 40 test tubes, and (D) garlic extract with 40 test tubes. Original images of study samples.

The study used 164 test tubes per trial, divided into four experimental groups (40 test tubes each) and one control group (four test tubes). The experimental groups consisted of 5.25% NaOCl (SafeEndo Hypochlor, Safe Endo Dental India Pvt. Ltd., Gujarat, India), 2% CHX (SafeEndo Hexachlor, Safe Endo Dental India Pvt. Ltd.), neem extract, and garlic extract. Each group was further subdivided into five subgroups based on irrigant volume (10, 20, 30, 40, and 50 µL), with eight test tubes per subgroup to ensure sufficient replicates (Figure 1).

The control group used 0.9% saline as the irrigant. The neem extract was prepared by boiling 100 g of fresh neem leaves (sourced locally) tied in a muslin cloth in 800 mL of distilled water until it was reduced to 400 mL (25% concentration), cooled, filtered through Whatman No. 1 filter paper (Whatman, Maidstone, UK), and stored in an amber bottle. Garlic extract was prepared by cleaning, peeling, and drying 100 g of garlic cloves, treating them with 70% ethanol (Merck, Darmstadt, Germany) for 60 seconds, evaporating residual ethanol in a laminar airflow chamber (Klenzaids, Mumbai, India), homogenizing using a sterile mortar and pestle, filtering through double-layer filter paper, and diluting to 25% with distilled water.

Data collection

Each test tube received 100 µL of diluted RBCs and the designated volume of irrigant (or saline for controls), followed by incubation at room temperature for three minutes to allow for interaction. After incubation, RBC viability was measured using an automated hematology analyzer (Diatron Abacus 380, DIATRON MI ZRT, Budapest, Hungary) calibrated before each use to ensure accurate hemoglobin content and hemolysis measurements. The analyzer’s calibration followed the manufacturer’s protocols to verify the precision.

The experiment was repeated eight times, totaling 1,312 test tubes (164 × 8), with the mean RBC viability calculated for each subgroup to account for variability. The discontinuation criteria included ineffective plasma separation during centrifugation, microbial contamination in samples or extracts, analyzer calibration errors, procedural deviations (such as incorrect irrigant volumes), and sample mishandling (such as spillage or improper labeling). Fresh samples were added to replace discarded samples to maintain sample size.

To ensure reliability, internal consistency was maintained by replicating the experiment eight times under identical conditions using standardized protocols for blood collection, processing, and analysis. Test-retest reliability was confirmed by consistent results across trials, and inter-rater reliability was achieved by training all personnel to adhere to uniform procedures. Internal validity was ensured by including a saline control group and randomizing the test tube allocation to isolate the irrigant effects. Content validity was supported by the use of established hemolysis measurement techniques, whereas construct validity was maintained through calibrated equipment and consistent methods, accurately reflecting irrigant-induced hemolysis. External validity was limited to in vitro settings and applicable to similar controlled conditions rather than clinical scenarios.

Statistical analysis

Data were analyzed using SPSS software version 20 (IBM Corp., Armonk, NY). Normality was assessed using the Shapiro-Wilk test, whereas trial consistency was evaluated using the intraclass correlation coefficient (ICC) analysis. As the data violated normality assumptions, non-parametric analyses were employed. Intergroup comparisons were performed using the Kruskal-Wallis test with post-hoc Bonferroni correction for multiple comparisons. Subgroup analyses were conducted using non-parametric methods. Statistical significance was set at p < 0.05 for all analyses.

## Results

All groups showed a statistically significant intraclass correlation (p < 0.05), with 5.25% NaOCl demonstrating near-perfect reliability (ICC = 0.99). The 2% CHX (ICC = 0.91) and garlic (ICC = 0.87) extract showed strong consistency, whereas the neem (ICC = 0.58) and saline control (ICC = 0.44) exhibited moderate reliability (Table 1).

**Table 1:** Trial consistency estimated with intraclass correlation analysis for study groups. * P < 0.05 denotes statistical significance. Test statistics are denoted as the F-value for intraclass correlation. NaOCl denotes sodium hypochlorite, and CHX denotes chlorhexidine.

Groups	Intraclass correlation coefficient	Lower limit at 95% confidence interval	Upper limit at 95% confidence interval	F value	p-value
5.25% NaOCl	0.99	0.98	0.99	639.4	0.001*
2% CHX	0.91	0.87	0.95	81.82	0.001*
Neem extract	0.58	0.46	0.71	12.18	0.001*
Garlic extract	0.87	0.81	0.92	54.74	0.001*
Saline (control)	0.44	0.09	0.93	10.56	0.004*

The control (saline) group showed the highest viability (median = 5.53%), followed by the neem (4.91%) and 2% CHX (4.82%) groups, while 5.25% NaOCl demonstrated the lowest RBC preservation (3.26%). Notably, herbal alternatives (neem and garlic) performed comparably to 2% CHX (p > 0.05), all showing significantly better RBC protection than 5.25% NaOCl (p < 0.001). These findings suggest that NaOCl has stronger cytotoxic effects, whereas herbal solutions may offer safer alternatives for clinical applications requiring RBC preservation (Table 2).

**Table 2:** Intergroup comparison of the percentage of viable human red blood cells (RBCs) among study groups using the Kruskal–Wallis test. Data are presented as median and mean ± standard deviation (SD). * P < 0.05 denotes statistical significance.

Groups	Median	Minimum	Maximum	Mean ± SD	Test statistics	p-value
5.25% sodium hypochlorite	3.26	1.94	4.47	3.34 ± 0.87	87.55	0.012*
2% chlorhexidine	4.82	3.90	5.16	4.60 ± 0.41		
Neem extract	4.91	4.57	5.21	4.90 ± 0.16		
Garlic extract	4.78	4.05	5.13	4.61 ± 0.34		
Saline (control group)	5.53	5.28	5.55	5.47 ± 0.13		

Post-hoc analysis with Bonferroni correction revealed significant differences in RBC viability between the multiple groups (p < 0.05). NaOCl showed significantly lower RBC preservation compared to all other groups (p < 0.01), including CHX, neem, garlic, and saline controls. While CHX demonstrated better RBC viability than NaOCl (p = 0.01), it showed no significant difference from that of garlic (p = 1.0). Notably, neem exhibited superior RBC protection compared with garlic (p = 0.04) and approached significance compared with saline (p = 0.072). The saline control group maintained the highest overall viability of RBCs (Table 3).

**Table 3:** Pairwise comparison using post-hoc analysis with Bonferroni test. NaOCl denotes 5.25% sodium hypochlorite, and CHX denotes 2% chlorhexidine. * Adjusted p < 0.05 denotes statistical significance. Significance is based on adjusted p-values to account for multiple comparisons.

Pairwise groups	Test statistics	Standard error	Unadjusted p-value	Adjusted p-value
NaOCl - CHX	-61.47	10.62	0.001	0.01*
NaOCl - Neem	-89.84	10.62	0.001	0.01*
NaOCl - Garlic	-59.44	10.62	0.001	0.01*
NaOCl - Saline (control)	-134.69	24.90	0.001	0.01*
CHX - Neem	-28.36	10.62	0.008	0.07
CHX - Garlic	2.04	10.62	0.848	0.99
CHX - Saline (control)	-73.21	24.90	0.003	0.03*
Neem - Garlic	30.40	10.62	0.004	0.04*
Neem - Saline (control)	-44.85	24.90	0.072	0.71
Garlic - Saline (control)	-75.25	24.90	0.003	0.03*

Kruskal-Wallis test revealed significant concentration-dependent effects on RBC viability across all groups. NaOCl showed the most dramatic decline in RBC preservation with increasing concentrations (median: 4.42% at 10% concentration vs. 1.96% at 50%). Although CHX, neem, and garlic maintained better RBC viability than NaOCl across concentrations, they also exhibited decreasing protection at higher concentrations. Notably, neem demonstrated the most stable RBC preservation, suggesting its superior cytoprotective properties (Table 4).

**Table 4:** Comparison of viable red blood cells percentage between subgroups within each group using the Kruskal–Wallis test. * P < 0.05 denotes statistical significance.

Groups	5.25% sodium hypochlorite		2% chlorhexidine		Neem extract		Garlic extract	
Subgroups	Median	Mean rank	Median	Mean rank	Median	Mean rank	Median	Mean rank
10% concentration	4.42	36.50	4.99	36.50	5.12	35.56	5.03	36.50
20% concentration	3.99	27.56	4.88	25.88	5.01	28.38	4.22	8.81
30% concentration	3.26	21.44	4.85	23.00	4.92	20.25	4.78	22.00
40% concentration	2.99	12.50	4.23	12.63	4.81	13.75	4.81	27.00
50% concentration	1.96	4.50	3.99	4.50	4.69	4.56	4.22	8.19
Test statistics	36.74		35.68		34.47		34.53	
p-value	0.001*		0.004*		0.013*		0.001*	

Post-hoc analysis revealed significant concentration-dependent effects for all agents (p < 0.05). For NaOCl, RBC viability decreased significantly at 10-40% (p = 0.001), 10-50% (p = 0.001), and 20-50% (p = 0.001) concentrations. CHX levels showed similar significant declines at 10-40% (p = 0.001), 10-50% (p = 0.001), and 20-50% (p = 0.003). Neem demonstrated significant reductions of 10-40% (p = 0.002) and 10-50% (p = 0.001). Garlic exhibited significant differences at 10-20% (p = 0.001), 10-50% (p = 0.001), 20-40% (p = 0.018), and 40-50% (p = 0.013). These findings demonstrated concentration-dependent cytotoxicity across all agents, with the most pronounced effects occurring at higher concentrations (40-50%), as shown in Table 5.

**Table 5:** Pairwise comparison using post-hoc analysis with the Bonferroni test for each group. * Adjusted p < 0.05 denotes statistical significance.

Groups	5.25% sodium hypochlorite		2% chlorhexidine		Neem extract		Garlic extract	
Pairwise comparison of different concentrations	Test statistics	Adjusted p-value	Test statistics	Adjusted p-value	Test statistics	Adjusted p-value	Test statistics	Adjusted p-value
10% vs. 20%	8.94	1.000	10.63	0.690	7.19	1.000	27.69	0.001*
10% vs. 30%	15.06	0.099	13.50	0.209	15.31	0.088	14.50	0.130
10% vs. 40%	24.00	0.001*	23.88	0.001*	21.81	0.002*	9.50	1.000
10% vs. 50%	32.00	0.001*	32.00	0.001*	31.00	0.001*	28.31	0.001*
20% vs. 30%	6.13	1.000	2.88	1.000	8.13	1.000	-13.19	0.239
20% vs. 40%	15.06	0.099	13.25	0.234	14.63	0.123	-18.19	0.018*
20% vs. 50%	23.06	0.001*	21.38	0.003*	23.81	0.001*	0.63	1.000
30% vs. 40%	8.94	1.000	10.38	0.758	6.50	1.000	-5.00	1.000
30% vs. 50%	16.94	0.037*	18.50	0.015*	15.69	0.073	13.81	0.180
40% vs. 50%	8.00	1.000	8.13	1.000	9.19	1.000	18.81	0.013*

The Kruskal-Wallis test revealed significant concentration-dependent effects on RBC viability for all tested agents on RBC viability (p < 0.001). NaOCl showed the most pronounced cytotoxic effects, with RBC preservation sharply decreasing from 4.42% (10% concentration) to 1.96% (50% concentration). CHX demonstrated moderate concentration sensitivity and better RBC protection than NaOCl at different concentrations. Notably, neem exhibited the most stable RBC preservation, with consistently higher mean ranks than those of the other groups. Garlic showed variable effects, performing comparably to CHX at lower concentrations, but demonstrating better preservation at higher concentrations (Table 6).

**Table 6:** Between-group comparison of viable red blood cells percentage at different concentrations using the Kruskal–Wallis test. * P < 0.05 denotes statistical significance.

Subgroups	10% concentration		20% concentration		30% concentration		40% concentration		50% concentration	
Groups	Median	Mean rank	Median	Mean rank	Median	Mean rank	Median	Mean rank	Median	Mean rank
5.25% sodium hypochlorite	4.42	4.50	3.99	4.56	3.26	4.50	2.99	4.50	1.96	4.50
2% chlorhexidine	4.99	17.13	4.88	20.69	4.85	20.94	4.23	12.63	3.99	12.50
Neem extract	5.12	25.94	5.01	28.31	4.92	25.88	4.81	23.81	4.69	28.50
Garlic extract	5.03	18.44	4.22	12.44	4.78	14.69	4.81	25.06	4.22	20.50
Test statistics	21.6		28.78		23.19		26.06		29.16	
p-value	0.001*		0.01*		0.002*		0.001*		0.012*	

Post-hoc analysis revealed concentration-dependent differences in RBC preservation between the agents. NaOCl consistently showed significantly lower viability than neem at all concentrations (p < 0.001) vs. CHX/garlic at lower concentrations (10-30%). At 40-50% concentrations, the NaOCl-garlic differences became significant (p < 0.01). CHX and neem showed comparable effects, except at 50% concentration (p = 0.004). Notably, neem outperformed garlic at 20% (p = 0.004), but showed similar effects at other concentrations. Garlic performance varied significantly with concentration for both NaOCl and CHX. These findings demonstrate that agent selection and concentration critically impact RBC preservation, with neem emerging as the most consistently protective option across the tested concentration ranges (Table 7).

**Table 7:** Pairwise comparison using post-hoc analysis with the Bonferroni test for each concentration. NaOCl denotes 5.25% sodium hypochlorite, and CHX denotes 2% chlorhexidine. * Adjusted p < 0.05 denotes statistical significance.

Pairwise comparison	10% concentration		20% concentration		30% concentration		40% concentration		50% concentration	
	Test statistics	Adjusted p-value	Test statistics	Adjusted p-value	Test statistics	Adjusted p-value	Test statistics	Adjusted p-value	Test statistics	Adjusted p-value
NaOCl - CHX	-12.62	0.042*	-16.12	0.003*	-16.44	0.003*	-8.12	0.496	-8.00	0.526
NaOCl - Neem	-21.44	0.001*	-23.75	0.001*	-21.37	0.001*	-19.31	0.001*	-24.00	0.001*
NaOCl - Garlic	-13.94	0.018*	-7.87	0.558	-10.19	0.179	-20.56	0.001*	-16.00	0.004*
CHX - Neem	-8.81	0.360	-7.62	0.623	-4.94	1.000	-11.19	0.101	-16.00	0.004*
CHX - Garlic	-1.31	1.000	8.25	0.470	6.25	1.000	-12.44	0.047*	-8.00	0.526
Neem - Garlic	7.50	0.657	15.88	0.004*	11.19	0.102	-1.25	1.000	8.00	0.526

## Discussion

The pronounced cytotoxicity exhibited by 5.25% NaOCl in this in vitro model aligns with its potent oxidative mechanism, in which it dissociates into hypochlorous acid and reactive oxygen species, inducing lipid peroxidation and protein chlorination in erythrocyte membranes [13]. This leads to osmotic imbalance and rapid hemolysis, as reactive intermediates disrupt hemoglobin stability and cellular integrity. Such effects are well documented in the endodontic literature, where NaOCl's tissue-dissolving capacity, which is beneficial for microbial elimination, poses risks to vital cells through non-specific oxidation [14]. In contrast, the relatively mild impact of 2% CHX stems from its cationic bisbiguanide structure, which electrostatically binds to anionic sites on cell surfaces, compromising membrane permeability without extensive oxidative damage [8]. This selective disruption explains CHX's intermediate position, as it affects RBC viability through leakage of intracellular components rather than wholesale destruction, which is consistent with reports of dose-related lysis in blood cell models.

Herbal alternatives, such as neem extract, demonstrated superior RBC preservation, attributable to its bioactive compounds, such as azadirachtin and nimbidin, which confer antimicrobial efficacy via bacterial membrane disruption while exerting antioxidant and anti-inflammatory effects on host cells [15]. Neem's polyphenolic content scavenges free radicals, mitigating oxidative stress that would otherwise exacerbate hemolysis, thus positioning it as a cytoprotective option [16]. This is corroborated by endodontic investigations highlighting neem biocompatibility, where natural alkaloids reduce cellular apoptosis compared to synthetic agents, potentially due to modulated inflammatory pathways [17]. Similarly, the performance of garlic extract reflects the dual role of its organosulfur constituents, notably allicin, which inhibits microbial enzymes through thiol group interactions but exhibits concentration-dependent hemolytic potential on RBCs via membrane pore formation [18]. However, at diluted levels, garlic's antioxidant properties, enhanced in aged preparations, counteract this by stabilizing erythrocyte enzymes such as catalase, explaining its comparability to CHX [18]. A previous study on garlic in dental applications underscores this balance, as its immunomodulatory effects limit cytotoxicity while maintaining antibacterial potency [11].

The concentration-dependent increase in cytotoxicity across all irrigants arises from increased molecular exposure, amplifying reaction kinetics as higher volumes facilitate greater diffusion into RBC membranes, intensifying damage thresholds [4]. According to a previous study, the cytotoxic effects of NaOCl and CHX are time- and dose-dependent. For NaOCl, this manifests as exponential hemolysis due to cumulative oxidative burden, while CHX's effects plateau at the saturation of binding sites [19]. Pashley et al. [20] documented that complete hemolysis of erythrocytes occurred following exposure to 5.25% NaOCl, even at a dilution of 1:1,000. Additionally, NaOCl has been shown to influence the protein constituents of blood, as well as the protein supernatants, which exhibit a tendency to decompose with an increase in NaOCl concentration [21].

Herbal extracts show shallower declines, likely owing to their self-limiting bioactive profiles, in which excess compounds may aggregate without proportional toxicity. This pattern mirrors broader endodontic research, where irrigant dilution modulates cellular viability, emphasizing the need for optimized protocols to minimize harm [15-18]. Notably, neem stability across concentrations suggests inherent buffering mechanisms, such as flavonoid-mediated cytoprotection, differentiating it from the variable response of garlic, which may involve allicin degradation at higher dilutions [15]. Arévalo-Híjar et al. [22] reported no toxicity of neem extracts at low concentrations.

These mechanistic insights underscore the inherent trade-offs in irrigant selection: while synthetic agents like NaOCl provide superior antimicrobial efficacy, this often comes at the expense of biocompatibility, whereas herbal alternatives, through their natural synergistic effects, offer a safer biological profile. Previous studies comparing herbal and chemical irrigants in various cell lines reinforce this, showing reduced apoptotic markers with neem and garlic, potentially via nuclear factor kappa-light-chain-enhancer of activated B cells (NF-κB) pathway inhibition [11-13,23]. The reliability variations, with NaOCl's high ICC reflecting consistent aggressive action versus neem's moderate consistency, possibly due to batch variability in phytochemicals, further underscore the need for standardized herbal preparations.

In clinical endodontics, these findings advocate the integration of neem and garlic extracts as adjuncts or alternatives to NaOCl and CHX, particularly in regenerative procedures or cases with periapical involvement, where RBC preservation is crucial to avoid complications such as extravasation-induced necrosis. By reducing cytotoxicity, herbal medicines could enhance healing outcomes, minimize postoperative inflammation, and support minimally invasive therapies, aligning with the shift toward biocompatible materials. Dilution strategies may further optimize safety and enable tailored irrigation protocols for vulnerable patients.

The in vitro nature of this study limits the extrapolation to in vivo dynamics, where tissue buffers and vascular clearance might attenuate these effects. Reliance on the blood of a single volunteer introduces genetic bias, potentially overlooking inter-individual variability in RBC resilience. Focusing on RBCs neglects the impact on other periapical cells, such as fibroblasts, and herbal extract preparations may vary in potency due to sourcing inconsistencies. Future studies should incorporate multi-donor samples, in vivo models, and long-term biocompatibility assays to validate these observations.

## Conclusions

This study demonstrated that 5.25% NaOCl exhibited the highest cytotoxicity against human RBCs in an in vitro setting, significantly reducing RBC viability compared to 2% CHX, neem extract, garlic extract, and the saline control. Neem extract has emerged as the most biocompatible irrigant, offering superior RBC preservation across various concentrations, closely followed by garlic extract and CHX, which perform comparably. These herbal irrigants present promising alternatives to synthetic agents, particularly NaOCl, for endodontic applications that require reduced cytotoxicity. The concentration-dependent cytotoxicity observed across all irrigants underscores the importance of optimizing irrigant volumes to balance antimicrobial efficacy with cellular safety, with the neem extract showing the most stable cytoprotective profile. These findings support the potential integration of neem and garlic extracts into endodontic therapy as biocompatible options, warranting further in vivo studies to confirm their clinical efficacy and safety.
